# Estimates of the value of life lost from COVID-19 in Ohio

**DOI:** 10.2217/cer-2020-0245

**Published:** 2021-02-17

**Authors:** Peter J Mallow

**Affiliations:** ^1^Health Services Administration, Xavier University, Cincinnati, OH 45207, USA

**Keywords:** coronavirus, COVID-19, Ohio, value of statistical life, years of potential life lost

## Abstract

The economic burden of mortality due to the novel coronavirus (COVID-19) extends beyond the lives lost. Data from the Ohio Department of Public Health and Social Security Administration was used to estimate the years of potential life lost, 72,274 and economic value of those lost lives, US$17.39 billion. These estimates may be used to assess the risk-trade off of COVID-19 mitigation strategies in Ohio.

We have long recognized that the burden of a disease (i.e., the novel coronavirus [COVID-19]) extends beyond the number of individuals infected or who die [1]. Thus, we need to understand the economic value of those premature deaths as a society to fully inform policy decisions about different mitigating strategies [1,2]. It has been estimated that mortality from COVID-19 has cut short approximately 2.5 million years of potential life in the USA [3]. However, the economic value of these lost life years was not estimated in this prior study, nor were local estimates of the years of potential life lost (YPLL) provided to aid state level policymakers, who have taken the lead in responding to COVID-19 [4]. This present study seeks to build upon this prior work to estimate the economic consequences of COVID-19 at the state level.

COVID-19 has contributed to 6,429 Ohioans deaths as of 30 November 2020 [5]. However, the full burden caused by COVID-19 mortality is not captured solely by the lives lost. Each death represents some number of YPLL had the individual not contracted COVID-19. Further, the statistical value of those lives lost (VSL) is not captured in mortality data alone. The estimated YPLLs and associated economic value for Ohio were estimated using data from the Ohio Department of Health and United States Social Security Administration [5,6]. The results suggested Ohio suffered 72,274 YPLLs and an associated lost VSL of US$17.39 billion dollars (Table 1).

**Table 1.** Years of potential life lost & value of statistical life.

	YPLL	VSL
Estimate		
Low	56,495	US$13,596,971,366
Base case	72,274	US$17,394,532,987
High	94,065	US$22,639,178,313

The Base case is the best estimate of YPLL and associated value of statistical life years lost. The low and high represent the estimated range.

VSL: Value of statistical life; YPLL: Years of potential life lost.

## Materials & methods

Ohio COVID-19 mortality was downloaded on 30 November 2020 from Ohio Department of Public Health’s (ODPH) website [5]. The data included all known cases of COVID-19 including hospitalizations and deaths by county of residence, age range and sex of the individual. The data are updated daily and changes often occur to past data based upon new information received by the ODPH. The age ranges were: 0–19, 20–29, 30–39, 40–49, 50–59, 60–69, 70–79 and 80+. The mid-point of the range was used to impute the age of death and the low- and high-end of the range was used to inform the sensitivity analysis. For those 80+, the mid-point was assumed to be 87 and the high-end to be 95. There were three deaths of unknown age that were removed from the analysis of YPLL and associated economic burden.

Actuarial life tables were downloaded from the Social Security Administration (SSA) to determine the remaining years of expected life by age [6]. The life tables provided estimates of remaining life expectancy by age and gender. The most recent life tables were published in 2017. The SSA used data published in the volumes of Vital Statistics of the United States and tabulated by the National Center for Health Statistics [7].

The YPLLs were calculated by subtracting the expected life expectancy from the imputed age of death by individual. The average value of a life year lost was US$247,676 and was obtained from previous published literature accounting for age [8]. The value of a life year lost, referred to as value of statistical life year (VSLY), is an estimate of the society’s willingness-to-pay for one year of life, and is used by state and federal policymakers to quantify the burden of disease or policy [1]. The VSLY was multiplied by the YPLL to determine the economic burden associated with the lost value of life (VSL) for each COVID-19 death. The YPLL and VSL results were summed to generate aggregate results for the state, gender and age (less than 60 & 60+).

## Results

**Figure 1.**
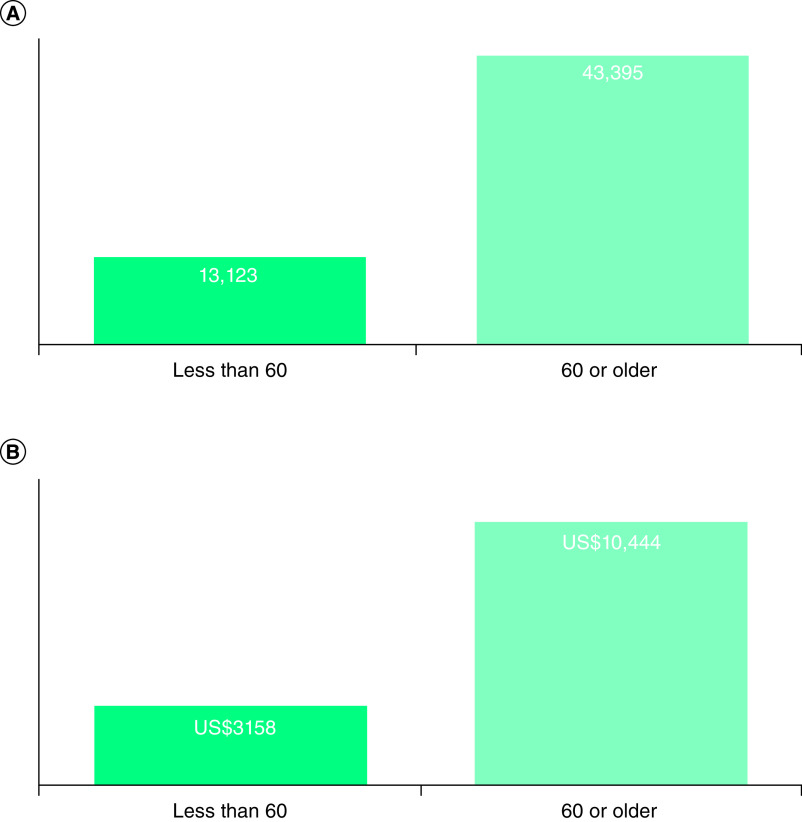
Years of potential life lost by age. **(A)** Estimated number of potential years of life lost by those under 60 years of age and those 60 and older; **(B)** value of the lost years of life by those under 60 years of age and those 60 years of age and older (in billions). VSL: Value of statistical life.

The 6,429 deaths in Ohio resulted in an expected YPLL of 72,274 (range: 56,495–94,065). The corresponding VSL was US$13.60 billion (range: US$10.39–US$17.98 billion) (Table 1). Based on the age of the COVID-19 deaths, the average loss of potential life years was 11.2 per person (range: 8.6–14.8 years). The breakdown by gender was 26,327 and 30,192 YPLLs for females (2478 deaths) and males (2600 deaths), respectively. The economic loss by gender was $6.34 (female) and 7.27 (male) billion (Table 2). Estimates by age indicated that 13,123 of the 56,518 YPLLs and US$3.16 billion VSL resulted from COVID-19 deaths under 60 (Figure 1). For those deaths under the age of 60, the average loss of potential life years was 31.0-person years (range: 27.6–35.3). Whereas, the YPLL in deaths aged 60 or greater was 9.4-person years (range: 6.8–13.0).

**Table 2.** Sex and gender breakdown of deaths, years of productive life lost & value of statistical life.

	Deaths	YPLL	VSL
Sex			
– Female	3109 (48%)	33,230 (46%)	US$7,997,547,956 (46%)
– Male	3302 (51%)	38,944 (54%)	US$9,372,886,144 (54%)
– Unknown	18 (<1%)	100 (<1%)	US$24,098,887 (<1%)
Age			
– Less than 60	522 (8%)	16,179 (22%)	US$3,893,834,428 (22%)
– 60 or older	5904 (92%)	56,089 (78%)	US$13,499,280,978 (78%)
– Unknown	3 (<1%)	NA	NA

The three deaths with unknown age were not included in the estimates of YPLL or the value of statistical life years lost.

NA: Not applicable; VSL: Value of statistical life; YPLL: Years of potential life lost.

## Discussion

Building upon the work conducted for the nation, this study estimated the YPLL and VSL due to COVID-19 in Ohio [3]. Assuming a consistent value of a year of lost life, the economic burden of premature deaths was estimated at $17.39 billion as of 30 November 2020. This value and the estimated YPLLs are rapidly changing with the current spread COVID-19 in the community. Thus, the results likely underestimate the YPLLs and VSL of the loss of life from COVID-19 in Ohio.

Separating the results by gender indicated that the age distribution and corresponding YPLLs and VSL were similar (48.4% female; 52.6% male). An examination by age indicated the disproportionate effect of those under COVID-19 deaths under 60 has on the expected overall results. Of the 6429 deaths, 522 (8.1%) were under 60. However, these deaths represented 22.4% of the total YPLLs and VSL. To date COVID-19 mortalities under 60 have been relatively few. However, the much greater expected loss of potential life years, average of 31.0-person years, contributed to the higher YPLL and VSL.

The method of estimating the VSL for a particular health condition is not without controversy [8,9]. The methodology used assumed a standard value of life across all individuals. As a result, allocation and equity issues associated with the assessment of differing policies may vary by age, income and race not accounted for in the results. Comparisons across diseases or geographic units should be made with great caution ensuring the underlying data and methods are consistent. Thus, this approach should be one of several when assessing strategies to mitigate the deleterious effects of COVID-19. Further, the ODPH does not release the specific age of the individuals who have died from COVID-19, which resulted in imputations to estimate the YPLLs and VSL. This limitation was mitigated by sensitivity analysis examining the low- and high-end of the age ranges. Finally, there is no generally accepted appropriate VSLY. The use US$247,676 VSL in this study was transparent and may easily be updated with differing amounts based upon the policymaker’s own determination.

These results have implications for state level policies addressing the COVID-19 pandemic. The estimates identified in this study for Ohio may provide a common and consistent framework to estimate the costs and benefits of differing COVID-19 mitigation strategies, such as vaccines, and novel treatments for COVID-19 or host response to the disease [1].

The recent announcement of two vaccines showing great promise regarding safety and efficacy raises the issue of who will receive the vaccine first [10]. The use of YPLLs may be one measure to assess the distribution of a vaccine within the state of Ohio and to whom should receive it first. As shown in the results, the burden of COVID-19 mortality is disproportionately higher in those under 60. The results of this study suggest prioritizing at-risk individuals under 60 in the vaccine distribution may substantially decrease premature mortality and increase economic productivity of the state.

## Conclusion

The expected loss of life-years from premature deaths of COVID-19 in Ohio was substantial. Each death represents a number of life-years lost and the enjoyment of those years with family and friends. Further, there is a substantial economic loss to all Ohioans. Estimating the economic burden of COVID-19 may provide a consistent means for state level policymakers to assess interventions control and mitigate the effects of the COVID-19 pandemic.

**Summary points**

- The novel coronavirus pandemic (COVID-19) has greatly increased mortality.
- Mortality attributable to COVID-19 disproportionately occur in individuals over the age of 60.
- Examining mortality by age only does not convey the full burden of COVID-19.
- The use of years of productive life and value of statistical life are two additional measures that combined with mortality may explain more of the burden related to COVID-19.
- The years of potential life lost and value of statistical life were estimated for the state of Ohio to illustrate the burden of COVID-19.
- This study found a disproportionate share of the burden related to COVID-19 occurs in those under 60 years of age.
- Policymakers may consider this information when determining how to allocate scarce resources (i.e., vaccine) for the treatment of COVID-19.
- Caution should be taken when reviewing these findings as they are only two data points in a multi-dimensional problem of allocation scare resources to address COVID-19.
